# Randomized controlled trial of procedural sequence for same-day bidirectional endoscopy under monitored anesthesia care (RECoVER Trial)

**DOI:** 10.1016/j.igie.2023.07.014

**Published:** 2023-07-25

**Authors:** Ali El Mokahal, Halim Bou Daher, Rana Yamout, Nour Hoshaimi, Chakib Ayoub, Yasser Shaib, Ala I. Sharara

**Affiliations:** 1 Division of Gastroenterology, Department of Internal Medicine; 2 Department of Anesthesia and Pain Medicine, American University of Beirut Medical Center, Beirut, Lebanon

## Abstract

**Background and Aims:**

Evidence regarding the ideal endoscopy sequence is inconclusive in patients undergoing same-day bidirectional endoscopy (BDE).

**Methods:**

Adults undergoing endoscopy with moderate sedation were randomized to either colonoscopy first (C-E) or EGD first (E-C) under monitored anesthesia care and bispectral index (BIS) monitoring. The primary endpoint was time to recovery from sedation. Secondary endpoints were total amount of sedative, time in deep sedation (defined as BIS <60), patient and endoscopist satisfaction, and adverse events. Patients were contacted the following day for assessment of postdischarge events including delayed recovery and effects on cognitive function and memory.

**Results:**

One hundred twenty consecutive patients were enrolled (60 per arm). Baseline characteristics were similar. There was no difference between the C-E and E-C groups in time to recovery (20.2 ± 8.8 minutes vs 17.9 ± 8.8 minutes), dose of propofol (219.9 ± 79.6 mg vs 217.6 ± 79.1 mg), total time spent in deep sedation (7.2 ± 6.6 minutes vs 9.5 ± 7.9 minutes), patient and endoscopist satisfaction, adenoma detection rate (38.3% vs 31.7%, *P* = .444), intraprocedural adverse events, or immediate adverse events. Time to reach the cecum was shorter for E-C patients (5.9 ± 3.5 minutes vs 7.3 ± 3.78 minutes, *P* = .033), whereas C-E patients reported higher rates of postdischarge dizziness (17.5% vs 3.5%, *P* = .015).

**Conclusions:**

There are no clinically significant differences between procedural sequences in same-day BDE. Decisions regarding the preferred sequence may be individualized, taking into consideration procedural yield, individual risk, and other factors such as the potential for aerosol generation and staff exposure. (Clinical trial registration number: NCT04096339.)

Same-day bidirectional endoscopy (BDE) is often done for patients with iron deficiency anemia, unexplained abdominal pain, and visible or occult GI bleeding.^1^^,^^2^ BDE can also be done for patients who have separate concomitant indications for both EGD and colonoscopy. An estimated 10% of all patients who undergo endoscopic procedures receive BDE.^3^ Performing both procedures on the same day has been shown to reduce the amount of anesthetic, recovery time, procedure time, and costs compared with completing procedures on separate occasions.^4^

Despite its convenience, there is no established sequence for BDE, and the sequence is determined by provider preference. Multiple BDE sequence trials have been done assessing different facets of endoscopy (Table 1), including the amount of sedation, time to recovery, adverse event rate, outcomes of endoscopy, and patient and provider satisfaction. With respect to endoscopy outcomes and adverse events, Choi et al^5^ found no differences in adenoma detection rates, colonoscopy completion, or difficult cecal intubation rates. However, Cho et al^6^ found that the quality of EGD was higher when EGD was done first. Sayın et al^7^ showed no difference in the time needed to reach the cecum but did find a higher rate of adverse events in the EGD first and then colonoscopy (E-C) sequence, but another study by Chen et al^8^ found similar rates of adverse events with both sequences.

**Table 1:** Literature review of previous randomized controlled trials

Study	Study design	No. of patients	Sedation type	Outcomes assessed	Results
Cho et al (2010)^6^	Randomized prospective study	80	Limited (no benzodiazepine or propofol)	Procedure quality Patient comfort	Quality of EGD is better when EGD is performed first Decreased discomfort with EGD first
Hsieh et al (2011)^9^	Randomized prospective study	176	Lidocaine spray, meperidine, propofol	Quantity of propofol used Patient comfort Recovery time Procedure time	Total propofol used was less when EGD was done first Colonoscopy was better tolerated when EGD was done first No effect on procedure time, recovery time, or postprocedure discomfort
Choi et al (2013)^5^	Randomized prospective study	1103	568 patients underwent sedation with midazolam and propofol	Colonoscopy completion rate Adenoma detection rate	No difference in colonoscopy completion rate No difference in difficult cecal intubation rates No difference in adenoma detection rates More subjective discomfort when colonoscopy was done first
Carter et al (2014)^14^	Randomized prospective study	163	Meperidine and 2.5 mg midazolam	Amount of sedation required to keep patient comfortable Postprocedure satisfaction	No difference in amount of sedation required No difference in postprocedure satisfaction
Tang et al (2016)^10^	Crossover study: patients completed C-E sequence initially, then E-C sequence 1.5 y later	63	Midazolam and fentanyl	Sedative dose Recovery time	Lower dose of fentanyl and midazolam with E-C sequence Shorter recovery time with E-C sequence
Cao et al (2017)^11^	Randomized prospective study	209	1 μg/kg remifentanil and 2 mg/kg propofol. additional doses of propofol (.5 mg/kg) were given during EGD or colonoscopy for escalating the sedation	Total propofol dose Recovery time Duration of endoscopy Patient satisfaction Adverse effects Endoscopic findings Cardiopulmonary responses of the patients	Less recovery time in E-C sequence E-C sequence had a lesser decrease in mean arterial pressure Less propofol during E-C sequence No difference in satisfaction of patients or physicians between the groups No difference in adverse effects in either group No difference in pathologic findings
Sayın et al (2020)^7^	Randomized prospective study	80	Fentanyl and propofol	Total propofol consumption Retching during procedure Time to cecum Endoscopist and patient satisfaction	More propofol required in E-C sequence Total procedure duration and EGD duration longer in E-C sequence Higher adverse event rate in E-C sequence Lower endoscopist and patient satisfaction in E-C sequence No difference in time to reach cecum
Chen et al (2018)^8^	Randomized prospective study	120	Midazolam and fentanyl	Total sedation used Recovery time Discomfort Adverse events	Mean dose of fentanyl and midazolam were higher in the C-E group Aldrete scores at 15 and 25 min were higher in the E-C group No difference in discomfort No difference in adverse events
Hammami et al (2019)^13^	Randomized prospective study	100	Conscious sedation (14%): fentanyl and midazolam Deep sedation: propofol infusion	Mean difference in procedure time Difference in medication doses	No difference in procedure time No difference in sedation doses
Jowhari et al (2020)^12^	Randomized prospective study (randomized sequence and randomized to CO_2_ vs air insufflation)	200	Midazolam/fentanyl	Comfort scores Amount of sedative required	E-C group required less midazolam Discomfort did not differ according to sequence on days 0 and 7 postprocedure

*E-C*, EGD first followed by colonoscopy; *C-E*, colonoscopy first followed by EGD.

In studies where quantity of sedation was assessed, 5 studies showed that the E-C sequence required less sedation.^5^^,^^8^, ^9^, ^10^, ^11^, ^12^ Two studies were done with light sedation, whereas other studies used propofol, showing more sedation in the colonoscopy and then EGD (C-E) sequence in 1 study^7^ and no difference in quantity of sedation in the other.^13^ Some studies have shown that the E-C sequence is associated with less patient discomfort,^5^^,^^6^^,^^9^ whereas 2 studies showed no difference in patient discomfort.^8^^,^^12^ Sayın et al^7^ reported that both patients and providers were less satisfied with the E-C sequence, and 3 other studies showed no difference in postprocedure satisfaction.^8^^,^^12^^,^^14^ Of the studies that assessed recovery time, 3 reported faster recovery with the E-C sequence,^8^^,^^10^^,^^11^ of which only 1 study used deep sedation with propofol. On the other hand, 2 other studies showed no difference in patients undergoing deep sedation.^9^^,^^13^

The bispectral index (BIS) is a noninvasive tool that uses electroencephalogram monitoring to provide anesthesiologists with a numerical representation of the depth of sedation and effects of anesthesia.^15^^,^^16^ The American Society of Anesthesiologists defines 4 levels of sedation: minimal, moderate, deep sedation, and general anesthesia. The corresponding BIS values, ranging from 100 to 0, are as follows: 100 to 95, awake; 95 to 70, light to moderate sedation; 70 to 60, deep sedation with low probability of explicit recall; 60 to 40, general anesthesia with low probability of consciousness; and <40, deep hypnotic state. When patients receive general anesthesia, their sedatives are usually titrated to maintain a BIS value of 40 to 60.^17^ As such, this tool can be used to identify states in which patients are oversedated. The harms of oversedation include a higher risk of hemodynamic instability, delayed recovery, and potential for delayed effect on cognitive function.

Given the heterogeneity among existing studies, we conducted a randomized controlled trial to assess the effect of procedural sequence on endoscopy parameters (RECoVER: Reduction in sEdation: Colonocopy Vs Esophagoduodenoscopy first). Additionally, our study is the first to directly assess the effect of procedural sequence on oversedation, as detected by BIS monitoring.

## Methods

### Study population

**Figure 1:**
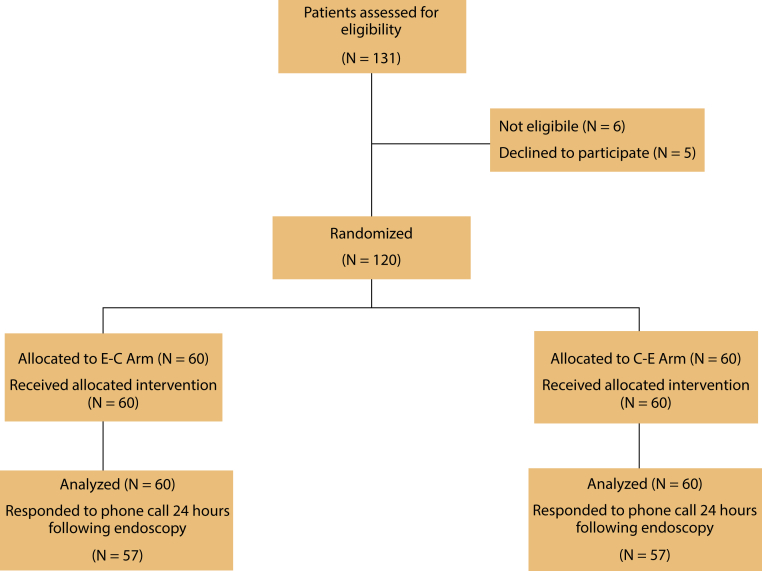
Flowchart describing patient enrollment in the study. *E-C*, EGD first followed by colonoscopy; *C-E*, colonoscopy first followed by EGD.

This was a randomized controlled trial conducted at the American University of Beirut Medical Center in Lebanon. Patient enrollment is described in Figure 1. Outpatients undergoing elective BDE were included if aged >18 years and scheduled for EGD and colonoscopy in the same session with anesthesiologist-administered sedation. Exclusion criteria were age <18 or >75 years; known allergy or adverse reaction to propofol, midazolam, or opioid medication; sleep apnea; American Society of Anesthesiologists class >III; pregnancy; known cirrhosis; chronic kidney disease (stage 4 or 5); known psychological disorder or cognitive dysfunction; significant gastroparesis; gastric outlet obstruction; ileus; known or suspected bowel obstruction; presence of a stoma; compromised swallowing reflex or mental status; prior colon resection or gastric surgery; and chronic use of >1 psychoactive drug (benzodiazepines, antidepressants, antipsychotics).

Informed consent was obtained from all enrolled patients. The study was approved by the institutional review board. The trial was registered at clinicaltrials.gov on September 19, 2019.

### Primary and secondary outcomes

The primary outcome of this study was time to recovery from sedation. Secondary outcomes were propofol requirements; depth of anesthesia using the BIS scale; cognitive function impairment up to 24 hours after discharge; duration of induction; biopsy sample kit use; hemodynamic instability; episodes of desaturations, apnea, drowsiness, and impaired cognitive function; and need for airway support.

### Sample size and data analysis

Our sample calculation was based on similar study results concerning recovery time. We estimated the subjects undergoing EGD first to have a shorter recovery period by almost 25%.^8^ Using patient recovery time as a primary endpoint, with an α of .05 and a power of .80, we calculated the sample required to show significance to be 51 patients per arm, assuming noninferiority. Assuming a 10% dropout, we aimed to enroll 120 subjects.

### Study design

Patients enrolled were divided into 2 groups: E-C sequence or C-E sequence. Patients were randomized using an online randomization tool that was not accessible by the endoscopists. Two senior gastroenterologists performed the procedures for this study. The endoscopists perform 1000 to 1200 endoscopic procedures per year and have 20+ and 25+ years of experience within the field.

After enrollment, patients were evaluated by the anesthesia team and prepared for endoscopy. Seven attending physician anesthesiologists provided the sedation for the procedures, each with 5+ years of work experience. Before induction, the BIS electroencephalogram probes were placed on the patient’s forehead, and the machine was turned on. The providing endoscopists, anesthesiologists, and patients could not be blinded to the procedural sequence. Endoscopy began when the patient was sedated on assessment and with BIS values between 70 and 80.

Time of initiation of induction and the initial doses of propofol, midazolam, and fentanyl were recorded. Time of scope insertion and scope removal were recorded for both procedures. At the end of the procedures, the time for transfer to the postanesthesia care unit was recorded, and transfer was determined at the discretion of the anesthesiologist. During the procedure, BIS readings, heart rate, blood pressure, and oxygen saturation level were recorded at the start of sedation and every 5 minutes during the procedure. Adverse events of interest were desaturation requiring jaw thrust, desaturation requiring airway insertion, bradycardia requiring atropine, hypotension requiring neosynephrine or ephedrine, or patient movement or awakening requiring an anesthetic bolus.

After the completion of both procedures, the anesthesiologist determined the total amount of propofol, fentanyl, and midazolam used. Duration of induction and time to reach the cecum were recorded. After the procedure, endoscopist (quality sedation and bowel preparation) and patient satisfaction were assessed. The patient’s BIS recordings were exported from the machine onto a computer, and the mean BIS reading every minute per procedure was recorded.

To assess recovery time, the modified postanesthesia discharge scoring system was used. This scoring system uses vital signs, activity, presence of nausea or vomiting, pain, or surgical bleeding to provide patients with a value. The modified postanesthesia discharge scoring system has been shown to be adequate for use in patients undergoing endoscopic procedures.^18^ Patients with a value ≥9 were deemed ready for discharge. This was assessed by the research assistant every 5 minutes until 2 consecutive values ≥9 were recorded. The time to recovery was then documented by calculating the difference in the time from transfer to the postanesthesia care unit to the time the patient was deemed stable for discharge. Approximately 24 hours after procedures, patients were called and asked about their day after their procedure, their comfort and level of function, and their overall satisfaction with the procedure and sedation.

### Sedation regimen

Propofol at a dose of 100 to 150 μg/kg/min was used according to patient weight and was delivered using an automated pump (Alaris GH Syringe Pump; BD, Eysins, Switzerland). In addition, patients received a standard dose of 50 μg fentanyl and 2 mg midazolam intravenously. A trained anesthesiologist provided sedation during the procedure for all patients. Patients were given boluses of propofol or fentanyl if they were awakening or moving during the procedure, and these boluses were recorded if given.

### Data analysis

The primary endpoint of this study was time to recovery. Secondary endpoints were the amount of propofol used, time spent with BIS score <60 and the number of times the patient’s BIS score dipped below 60, cognitive impairment 24 hours after discharge, duration of induction, hemodynamic instability, episodes of desaturations or apnea, or need for airway support.

Continuous variables are expressed as mean ± standard deviation (95% confidence interval). Categorical variables are represented as counts with percentages. Group comparisons of qualitative variables were performed using χ^2^ tests and analysis of variance tests/Fisher exact test where applicable. *P* values <.05 were deemed significant. IBM SPSS, version 24 (IBM SPSS Statistics for Windows, Armonk, NY, USA) was used for data analysis

## Results

Baseline demographics for the 2 groups were similar (Table 2). Thirty-four patients used alcohol occasionally, of which 16 (26.7%) were in the C-E group and 18 (30%) were in the E-C group. Thirteen patients (10.8%) used 1 psychoactive medication, of which 7 (11.6%) were in the C-E group and 6 (10%) were in the E-C group. Ninety percent of patients had a history of sedation with general anesthesia (75%), of which 44 (73.3%) were in the C-E group and 46 (76.7%) were in the E-C group.

**Table 2:** Baseline demographics of enrolled patients

	Colonoscopy first, then EGD sequence	EGD first, then colonoscopy first sequence	*P* value
	No. of cases (%)	No. of cases (%)	
Female sex	29 (48.3)	29 (48.3)	1.000
Smoker	21 (35.0)	20 (33.0)	.847
Alcohol occasionally	16 (26.7)	18 (30.0)	.734
Alcohol frequently	5 (8.0)	3 (5.0)	
Psychotropic drug use	7 (11.6)	6 (10.0)	.769
Opioid drug use	0	0	
Diabetes	7 (11.6)	6 (10.0)	.769
Prior abdominal surgery	24 (40.0)	25 (41.67)	.853
Previous general anesthesia	44 (73.3)	46 (76.67)	.673

	No. of cases	Mean	Standard deviation	95% Confidence interval	No. of cases	Mean	Standard deviation	95% Confidence interval	
Age, y	60	54.5	11.6	50.6-56.6	60	53.5	11.63	50.6-56.6	.719
Weight, kg	60	74.9	13.9	71.3-78.5	60	80.5	17.55	76.0-85.1	.055
Height, m	60	1.7	.09	1.6-1.7	60	1.7	.11	1.68-1.74	.196

### Postprocedure recovery from sedation and time with BIS <60

These findings are presented in Table 3. The primary endpoint, time to recovery, was not significantly different. Mean time to recovery was shorter in the E-C group (17.9 ± 8.8 minutes) compared with the C-E group (20.3 ± 8.8 minutes, *P* = .144). Time to discharge was also not significantly different in the E-C group (44.4 ± 22.3 minutes) compared with the C-E group (44.7 ± 16.6 minutes). The total procedure time was not significantly different in the C-E group (23.5 ± 5.2 minutes) compared with the E-C group (23.9 ± 6.2 minutes, *P* = .662).

**Table 3:** Recovery from sedation and time in deep sedation

	Colonoscopy first, then EGD sequence				EGD first, then colonoscopy first sequence				*P* value
	No. of cases	Mean	Standard deviation	95% Confidence interval	No. of cases	Mean	Standard deviation	95% Confidence interval	
Time to recovery, min	60	20.3	8.8	15.6-20.2	60	17.9	8.8	18.0-22.6	.144
Time from scope out to discharge, min	60	44.7	16.6	40.3-49.1	60	44.4	22.3	38.6-50.1	.934
Total procedure time, min	60	23.5	5.2	22.1-24.8	60	23.9	6.2	22.3-25.5	.662
Time spent with <60, min	45	7.2	6.6	5.2-9.2	50	9.5	7.9	7.2-11.7	.142
Time from scope out to ready for transfer to postanesthesia care unit, min	60	3.1	2.7	2.4-3.8	60	1.4	1.8	.9-1.9	<.001

No. of events	No. of cases (%)	No. of cases (%)	
Bispectral index dips <60	40 (88.9)	44 (88.0)	1.000
No dips <60	5 (11.1)	6 (12.0)	
Total	45 (100)	50 (100)	

Forty-four patients (88.9%) in the E-C group experienced dips in BIS <60 compared with 40 patients (88.9%) in the C-E group (*P* = .825). The time spent with BIS <60 was 2 minutes longer on average in the E-C group (9.08 ± 7.68 minutes) compared with the C-E group (7.06 ± 6.28 minutes), but this was not statistically significant (*P* = .142). There was a statistically significant difference in the time it took patients to be ready for transfer to the postanesthesia care unit after the end of the procedures. In the C-E sequence, the time was 3.1 ± 2.7 minutes compared with 1.4 ± 1.8 minutes for the E-C sequence (*P* = .015).

### Anesthetic usage

These findings are presented in Table 4. Total propofol use was similar in C-E group (219.9 ± 79.6 mg) and the E-C group (217 ± 79.1 mg, *P* = .679). The same induction doses of propofol were given to patients in the C-E group (37.1 ± 11.6 mg) and the E-C group (38 ± 14.2 mg, *P* = .505). There was no difference in the number of patients who required propofol boluses in the C-E group (33 [55.0%]) and the E-C group (26 [43.3%]), *P* = .201).

**Table 4:** Anesthetic usage

	Colonoscopy first, then EGD sequence				EGD first, then colonoscopy first sequence				*P* value
	No. of cases	Mean	Standard deviation	95% Confidence interval	No. of cases	Mean	Standard deviation	95% Confidence interval	
Total propofol used, mg	60	219.9	79.6	199.3-240.5	60	217.6	79.1	197.2-238.1	.679
Propofol dose given before procedures, mg	60	37.1	11.6	34.1-40.1	60	38.7	14.2	35.0-42.3	.505
Total fentanyl, μg	60	59.8	18.7	54.9-64.6	60	64.8	21.4	59.3-70.4	.080
Amount of fentanyl dose before procedures, μg	60	57.9	17.3	53.5-62.3	60	62.7	19.5	57.7-67.8	.153
Amount of midazolam dose before procedures, mg	60	1.9	0.6	1.7-2.0	60	2.8	0.6	1.9-2.2	.203

	No. of cases (%)	No. of cases (%)	
No propofol boluses required during the procedure	33 (55.0)	26 (43.3)	.201
Propofol boluses required during the procedure	27 (45.0)	34 (56.7)	
Total	60 (100)	60 (100)	
No fentanyl boluses required during the procedure	40 (66.7)	51 (85.0)	.019
Fentanyl boluses required during the procedure	20 (33.3)	9 (15.0)	
Total	60 (100)	60 (100)	
No propofol bolus between procedures	59 (98.3)	60	.315
Propofol bolus between procedures	1 (1.7)	0	
Total	60 (100)	60 (100)	
No fentanyl bolus between procedures	42 (70)	56 (93.3)	.001
Fentanyl bolus between procedures	18 (30)	4 (6.7)	
Total	60 (100)	60 (100)	

Total fentanyl use was higher in the E-C group (64.8 ± 21.4 μg) compared with the C-E group (59.8 ± 18.7 μg), but this was not statistically significant (*P* = .080). The induction dose of fentanyl was similar when comparing between the C-E (57.9 ± 17.3 μg) and E-C (61.9 ± 20.9 μg, *P* = .153) groups. A larger number of patients received fentanyl boluses in the C-E group (20 [33.3%]) compared with the E-C group (9 [15.0%], *P* = .019). This was mainly driven by fentanyl boluses given between procedures, which were given to 18 patients (30%) in the C-E group and 4 patients (6.7%) in the E-C group (*P* < .001).

Midazolam was only used for induction. Its use was similar in the C-E group (1.9 ± .6 mg) compared with the E-C group (2.8 ± .6 mg, *P* = .203).

### Endoscopic parameters

These findings are presented in Table 5. The adenoma detection rate was similar in the C-E (.38%) and E-C (.32%) groups (*P* = .444). The overall adenoma detection rate was 35%, showing a high quality of colonoscopy. Similar biopsy samples were taken in both EGD and colonoscopy in the E-C group (31 [51.7%] and 33 [55.0%], respectively) and in the C-E group (26 [43.3%] and 37 [61.7%], respectively). The time to reach the cecum was lower in the E-C group (5.9 ± 3.4 minutes) compared with the C-E group (7.3 ± 3.7 minutes, *P* = .033). Endoscopist satisfaction with sedation and bowel preparation were high and similar in both groups.

**Table 5:** Endoscopy outcomes

	Colonoscopy first, then EGD sequence		EGD first, then colonoscopy first sequence		*P* value
	Total	No. of cases (%)	Total	No. of cases (%)	
Adenoma detection rate	60 (100)	23 (38.3)	60 (100)	19 (31.7)	.444
EGD biopsy samples taken	60 (100)	26 (43.3)	60 (100)	31 (51.7)	.361
Colonoscopy biopsy samples taken	60 (100)	37 (61.7)	60 (100)	33 (55.0)	.459
Endoscopist 1	60 (100)	54 (90.0)	60 (100)	56 (93.3)	.509

	No. of cases	Mean	Standard deviation	95% Confidence interval	No. of cases	Mean	Standard deviation	95% Confidence interval	
Time to cecum, min	60	7.3	3.7	6.3-8.3	60	5.88	3.45	4.99-6.77	.033
Withdrawal time, min	60	11.6	5.7	10.1-13.0	60	11.4	4.6	10.2-12.6	.903
Endoscopist satisfaction with sedation (0-5 scale)	60	4.50	.87	4.28-4.71	60	4.60	.62	4.44-4.77	.447
Endoscopist satisfaction with bowel preparation (0-5 scale)	60	4.53	.80	4.32-4.73	60	4.52	.874	4.28-4.75	.961

### Endoscopy adverse events

These findings are presented in Table 6. Adverse event rates were low overall. No patients had bradycardia requiring atropine, and only 1 patient in the E-C group required airway support with a nasopharyngeal tube. Desaturations requiring jaw thrust were higher in the C-E group (20 [33.3%]) compared with the E-C group (17 [28.33%]), but this was not statistically significant (*P* = .510). The rate of hypotension requiring neosynephrine was the same in both groups (3 [5%]). Patient movement requiring a bolus was lower in the C-E group (26 [43.33%]) when compared with the E-C group (30 [50.00%]), but this was not statistically significant (*P* = .464).

**Table 6:** Endoscopy adverse events

	Colonoscopy first, then EGD sequence		EGD first, then colonoscopy first sequence		*P* value
	Total	No. of cases (%)	Total	No. of cases (%)	
Desaturation requiring jaw thrust	60 (100)	20 (33.3)	60 (100)	17 (28.3)	.553
Desaturation requiring airway	60 (100)	0 (0)	60 (100)	1 (1.7)	1.000
Bradycardia requiring atropine	60 (100)	0	60 (100)	0	
Hypotension requiring pressors	60 (100)	3 (5.0)	60 (100)	3 (5.0)	1.000
Patient movement requiring bolus	60 (100)	26 (43.3)	60 (100)	30 (50)	.464

### Assessment 24 hours postprocedure

These findings are presented in Table 7. Patients noted high rates of overall satisfaction with the procedure, and this was similar in the C-E and E-C groups. Adverse events in the 24 hours after the procedure were rare overall. Two patients in the C-E group reported memory problems compared with 3 in the E-C group (*P* = 1.000). Seven patients (12.3%) in the C-E group reported nausea compared with 4 patients (7.0%) in the E-C group (*P* = .341). Dizziness was higher in the C-E group, occurring in 10 patients (17.5%) compared with 2 patients (3.5%) in the E-C group (*P* = .015). Difficulty focusing was the most common adverse event, reported by 12 patients (21.1%) in the C-E group and 9 patients (15.8%) in the E-C group (*P* = .469). Despite all patients counseled not to drive after the procedure, 30 patients drove (25%), including 15 patients from each group. None of the patients surveyed reported any accidents or issues with driving.

**Table 7:** Assessment 24 hours postprocedure.

	Colonoscopy first, then EGD sequence			EGD first, then colonoscopy first Sequence			*P* Value
	Total	Yes No. of cases (%)	No No. of cases (%)	Total	Yes No. of cases (%)	No No. of cases (%)	
Memory problems	57	2 (3.5)	55 (96.5)	57	3 (5.3)	54 (94.7)	1.000
Nausea	57	7 (12.3)	50 (87.7)	57	4 (7.0)	53 (93)	.341
Dizziness	57	10 (17.5)	47 (82.5)	57	2 (3.5)	55 (96.5)	.015
Difficulty focusing	57	12 (21.1)	45 (78.9)	57	9 (15.8)	48 (84.2)	.469
Drove after procedure	57	15 (26.3)	42 (73.7)	57	15 (26.3)	42 (73.7)	.584

	No. of cases	Mean	Standard deviation	95% Confidence interval	No. of cases	Mean	Standard deviation	95% Confidence interval	
“How long did it take you to go back to normal after discharge,” h	57	3.80	7.66	1.76-5.83	57	1.91	4.9	.62-3.22	.122
Overall satisfaction with procedure	57	9.63	.587	9.48-9.79	57	9.54	.761	9.33-97.4	.450

## Discussion

In this randomized controlled trial of sequence in BDE, we found no meaningful difference in our primary endpoint, namely time to recovery from sedation. Other studies have shown conflicting results. Hsieh et al^9^ found no significant difference in recovery time between the 2 sequences. Others found that recovery was faster for the E-C sequence.^8^^,^^10^^,^^11^ In the studies by Tang et al^10^ and Chen et al,^8^ sedation was given using only midazolam and fentanyl, without propofol. Compared with other agents used, propofol is known to have shorter recovery times.^19^ It is plausible that the use of propofol for sedation may have eliminated any difference in time to recovery between the 2 sequences, as was seen in the studies using midazolam and fentanyl for sedation. Cao et al^11^ found a mean difference of 2.2 minutes in recovery. Because we assessed recovery every 5 minutes, our intervals of assessment may not have been precise enough to detect such a small difference. Additionally, Cao et al^11^ used intermittent boluses of propofol to maintain sedation, whereas we used a continuous propofol drip with boluses as needed. It is unclear if an anesthesiologist was present in their procedures, and the presence of trained anesthesiologists in our study may have contributed to the absence of a difference. In calculating the power required for our study, we also assumed a 25% difference in recovery times, and this study may not have been powered adequately to detect a smaller difference in time to recovery. We did find that recovery was 2.3 minutes longer with the C-E sequence when compared with the E-C sequence, and this difference may have clinical or financial implications for endoscopy unit throughput.

We also found no difference in the time spent in deep sedation or the number of dips into deep sedation. In our experience, the intubation phase of EGD is more uncomfortable than colonoscopy, and we speculated that performing the EGD initially may be optimal because the initial induction medications would remain in the body and that any additional anesthetic usage would also carry forward into the colonoscopy. This contrasts with beginning with colonoscopy where we expected the need for added sedation and more boluses to complete the EGD while maintaining patient comfort. This is reflected in our study with our higher use of fentanyl when transitioning from colonoscopy to endoscopy. However, this was not associated with a statistical or clinically significant difference in the time spent in deep sedation. The absence of a difference in deep sedation between the 2 procedures can also be explained by the fact that patients in both sequences required similar amounts of sedation.

The similar amount of anesthetic required in our study goes against the grain when compared with most other studies. Five studies found that the amount of sedation required was lower with the E-C sequence,^8^, ^9^, ^10^, ^11^, ^12^ whereas 1 study found no difference^13^ and 1 study found a larger amount was required when using the C-E sequence.^7^ Additionally, a meta-analysis of 4 of the 5 studies showing lower amounts of sedation required confirms this.^20^ This difference seems to persist regardless of whether midazolam or propofol was used as the sedative. We and others^8^^,^^11^ also did not find a significant difference in the rates of adverse events between the 2 sequences. As such, the clinical significance of a difference in sedation doses may be unlikely. Additionally, the studies that reported a statistically significant difference in the amounts of sedation used did not report a markedly large difference.^20^ We did, however, find a lower rate of postprocedural dizziness in the 24 hours after the procedure when EGD is done first. However, there were no differences in the rates of nausea, memory loss, or difficulty focusing, and overall satisfaction with the procedures was high irrespective of sequence. We are unsure what the cause of this discrepancy was, particularly when the amount of sedation and time to recovery were not different and there were no identified differences between the 2 cohorts at baseline. However, it may be reasonable to identify patients who report a predisposition or history of dizziness and to begin with endoscopy in that subset of patients.

Overall, we did not find an appreciable difference to justify 1 sequence over the other. With the myriad factors assessed in the literature, it is difficult to identify 1 sequence that is optimal in all scenarios, and as such the decision of which procedure is done first needs to be determined by the setting and the patient population in which the procedures are conducted. One major benefit of performing EGD first is that biopsy forceps used in EGD are reusable in colonoscopies, but the reverse is not possible. In our colonoscopy-first cohort, 26 of 60 patients required subsequent EGD biopsy sampling, and in all these cases new forceps had to be used. This can add significant costs to endoscopy, which may merit consideration in low-resource areas or areas in the world where biopsy forceps are harder to attain. Finally, since December 2019 the severe acute respiratory syndrome *coronavirus 2 (*SARS-CoV-2) virus led to a global pandemic with which we continue to grapple to this day.^21^ This pandemic put healthcare workers and their families at increased risk of contracting the virus.^22^ Although this risk has since been decreased by the advent of vaccines,^23^^,^^24^ 1 group at a higher risk of contracting the virus are those involved in aerosol-generating procedures. EGD is an aerosol-generating procedure,^25^ and aerosols containing SARS-CoV-2 have been shown to remain stable for 3 hours.^26^ Masking and universal precautions are of paramount importance in these circumstances, and given the absence of clinically significant differences between the 2 sequences, we recommend that in times where SARS-CoV-2 cases rise or in any future concern of an airborne epidemic that colonoscopy be done first to minimize the exposure time of healthcare providers to aerosolized infectious agents in the examination room.

This study had several limitations. First, neither the endoscopists nor anesthesiologists performing the procedures could be blinded to the sequence of endoscopic performance. Furthermore, the study was performed by 2 senior endoscopists, and it is unclear how generalizable our results may be to other providers. Additionally, this study only assessed patients undergoing sedation with propofol, and our findings may not be generalizable to other methods of procedural sedation.

In conclusion, we did not find clinically significant differences between the 2 sequences of BDE when we assessed time to recovery, quantity of sedative used, patient satisfaction, adverse events, or endoscopist satisfaction with the procedure. Additionally, we did not find any differences in oversedation as detected by BIS monitoring. One strength of this study is the novel use of BIS monitoring to detect differences in sedation. BIS monitoring provides an objective method to assess oversedation that is not subject to bias, particularly because the endoscopist and anesthesiologist could not be blinded to procedural sequence. It also allows us to detect subclinical oversedation. Although we did not find a difference in procedural sequence, this study also shows that oversedation does often take place in endoscopy, and further assessment of subclinical oversedation in future studies is warranted to decrease the risks of oversedation in endoscopy. Based on the above, the decision regarding preferred sequence may be individualized, taking into consideration procedural yield, individual risk, additional cost of accessories, and other factors such as the potential for aerosol generation and staff exposure.

## Disclosure

All authors disclosed no financial relationships.
